# Plant Genome Size Is Associated With Fine‐Scale Spatial Variation in Soil Depth, but Not Climatic Conditions, in the Grass *Festuca ovina*

**DOI:** 10.1002/ece3.72438

**Published:** 2025-11-27

**Authors:** Sarah Trinder, Oriane Hidalgo, Michael F. Fay, Andrew P. Askew, Hugh McAllister, Raj Whitlock

**Affiliations:** ^1^ Department of Evolution, Ecology and Behaviour, Institute of Infection, Veterinary and Ecological Sciences University of Liverpool Liverpool UK; ^2^ Jodrell Laboratory Royal Botanic Gardens, Kew Richmond Surrey UK; ^3^ Institut Botànic de Barcelona (IBB, CSIC‐CMCNB) Barcelona Catalonia Spain; ^4^ School of Plant Biology University of Western Australia Crawley Western Australia Australia; ^5^ Department of Biochemistry & Systems Biology University of Liverpool Liverpool UK

**Keywords:** climate change, drought, evolution, *Festuca ovina*, intraspecific variation, plant genome size, selection, soil depth

## Abstract

Genome size varies among individuals within plant species and their populations. Interspecific variation in plant genome size is associated with phenology, climate, and latitude and longitude—suggesting that genome size may be linked with environmental adaptation—but the evolutionary significance of intraspecific variation in genome size remains unresolved. In particular, little is known regarding how selection under different climatic and micro‐environmental conditions shapes intraspecific variation in genome size. We measured genome size within *Festuca ovina* populations collected from grassland plots exposed to 16 years of experimental drought treatment at the Buxton Climate Change Impacts Laboratory. We assessed whether genome size was associated with either drought treatment or fine‐scale heterogeneity in soil depth within grassland plots. Genome size varied by up to 1.28‐fold among *F. ovina* individuals, but was not associated with either drought treatment or plant phenotypes (cell size, flowering time, and biomass). Genome size was, however, negatively associated with fine‐scale variation in soil depth, implying that abiotic and biotic conditions linked with soil depth impose either direct or indirect selection on genome size. We suggest that the higher nutrient availability and reduced competition associated with shallow soils enable individuals with larger genomes to persist locally within the grassland.

## Graphical abstract

Intraspecific variation in plant genome size could allow adaptation to changing environmental conditions. Using the world's longest running drought manipulation experiment, we make a long‐term experimental test of the relationship between population‐level plant genome size and drought. We show that while plant genome size is not altered by drought treatment, it is associated with fine‐scale natural heterogeneity in soil depth.

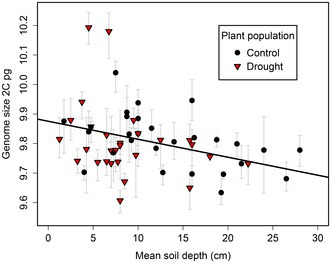

## 1 Introduction

Genetic variation underpins the ability of plant populations to adapt in response to global environmental change (Franks et al. 2014; Shaw and Etterson 2012; Trinder et al. 2020). Studies investigating climate‐driven evolutionary and genetic change in plants typically assume implicitly that genome size—the total amount of DNA in a cell nucleus—is constant for all individuals in a population (Franks et al. 2007; Ravenscroft et al. 2015). However, genome size in plants is known to vary widely between plant species, and both between and within plant populations (Balant et al. 2022; Leitch and Leitch 2013). This genome‐size variation is primarily the result of repetitive DNA, particularly transposable elements, which frequently account for more than half of the plant genome (Bennetzen et al. 2005; Meagher and Vassiliadis 2005), with smaller contributions from polyploidy and gene content or copy number (Gaut 2002). Genetic variation in genome size may be selectively non‐neutral (Kalendar et al. 2000). However, the extent to which genome size shapes (and is shaped by) adaptive and evolutionary responses to climate change remains poorly understood.

Plant genome size may be either adaptive or costly in its own right (hence a direct target of natural selection; Knight et al. 2005; Hessen et al. 2010), or could be genetically correlated with other traits that are themselves targets of selection, allowing genome size to respond indirectly to selection pressures (Beaulieu 2010). Two key hypotheses posit that the evolution of large genome size is associated with environmentally contingent costs to fitness, shaping the geographical distribution of plant species and the fitness of their individuals. Under the ‘large genome constraint’ hypothesis, the cost of maintaining additional repetitive DNA in the genome curtails speciation rates in large‐genome plant lineages, limits their occurrence in extreme environments and is associated with lower levels of plant performance (Knight et al. 2005). The ‘genome streamlining’ hypothesis (Hessen et al. 2010) predicts that large genomes will be selected against in environments that are both nutrient‐limited and require rapid or competitive growth, due to the high demand for nitrogen and phosphorus atoms for synthesis of DNA and RNA. The two hypotheses, therefore, differ in their prediction of the environments that select against individuals with large genome size. Under the large‐genome constraint hypothesis, all extreme or stressful environments, including those that are climatically stressful, could select against large genome size (Knight et al. 2005). In contrast, the specific conditions needed to select against plants with large genome size in the genome streamlining hypothesis are those where a requirement for rapid growth is combined with conditions of nutrient limitation. Evidence from nutrient manipulation experiments in grasslands has provided support for the latter hypothesis: plant communities that are limited by either or both N or P availability are characterised by species with smaller genome sizes, whereas large‐genome plant species are favoured when both elements are abundant (Guignard et al. 2016; Šmarda et al. 2013).

Species‐level estimates of genome size correlate predictably with plant phenotypes. For instance, it has been shown that nuclear DNA content is positively associated with cell volume and the duration of the cell cycle, placing ecologically relevant constraints on cell division and growth rates (Van't Hof and Sparrow 1963; Leitch and Leitch 2013). DNA content is also associated at the species level with seed mass (Beaulieu et al. 2007), specific leaf area (Knight et al. 2005) and phenology (Grime and Mowforth 1982; Grime et al. 1985). In addition to the expected direct cytological effects of genome size (Van't Hof and Sparrow 1963), plant phenotypes can also be altered by the proliferation of transposable elements through insertional mutations and changes to the regulation of gene expression (Meagher and Vassiliadis 2005; Lisch 2013). These genetic or regulatory changes may be adaptive, resulting in selection for their proliferation, and an associated increase in genome size (Casacuberta and González 2013; Kalendar et al. 2000). Genome‐size‐associated changes in plant phenotypes also shape critical aspects of plant life‐history strategy, including competitive ability and stress tolerance, linking genome size to ecological strategy (Knight et al. 2005; Guignard et al. 2016). The pervasive evidence for associations between plant genome size and phenotypes suggests that relationships between genome size and climate gradients should also exist.

Plant genome size is known to be associated at the species level with climatic factors (Bureš et al. 2022; Knight and Ackerly 2002; Macgillivray and Grime 1995; Schley et al. 2022), with altitude and with latitude (reviewed by Knight et al. 2005). For example, in the British flora, spring growth is negatively correlated with genome size (Grime and Mowforth 1982). Since cold temperatures limit cell division, species with large genomes are thought to carry out cell division in the previous summer. These species are then able to grow rapidly by cell expansion as water becomes available in early spring, allowing them to exploit a distinct phenological niche (Grime et al. 1985).

Only a small number of studies have addressed associations between intraspecific variation in genome size and plant phenotypes and environmental variables (reviewed in Greilhuber and Leitch 2013). At ‘Evolution Canyon’ (Israel), the relationship between genome size and contrasting microclimatic conditions on opposing slope aspects (drier south‐facing vs. wetter north‐facing aspects) has been investigated in four species (Nevo 2012). In two of these, *Ceratonia siliqua* and *Hordeum spontaneum* , plant genome size was greater in populations on the drier south‐facing slope when compared with the wetter north‐facing slope (Bureš et al. 2004; Kalendar et al. 2000). Additionally, in *C. siliqua*, genome size was significantly negatively correlated with leaf length and tree circumference (Bureš et al. 2004). Furthermore, in *H. spontaneum* , the highest copy number of the retrotransposon *BARE*‐1, which is positively correlated with genome size, was found at the most arid sites (Kalendar et al. 2000; Vicient et al. 1999). In temperate grasslands, genome size in *Festuca pallens* varies with latitude and longitude (and hence with climatic variation) across its geographical range (Šmarda and Bureš 2006), but is not associated with microclimatic conditions at fine spatial scales (Šmarda et al. 2007). Genome‐size variation within *F. pallens* has been linked to plant phenotypes, correlating with plant development rate (Šmarda et al. 2008), and is shaped by stabilising selection at the population level (Šmarda et al. 2008, 2010).

Collectively, these studies reveal a link between intraspecific variation in genome size, the climate and plant phenotypes and implicate transposable elements in driving intraspecific variation in genome size. However, a long‐term experimental test of the relationship between intraspecific variation in genome size and climatic conditions has not been undertaken. In addition, the mechanisms underpinning such a relationship, and whether genome size itself is the proximate target of natural selection, remain poorly understood (Beaulieu 2010; Šmarda et al. 2010).

The genus *Festuca* is an excellent model for understanding the role of climatic variation in shaping intraspecific variation in genome size: its species have a wide ecological breadth (Bradshaw and Snaydon 1959; Wilkins 1960), are known to possess variation in genome size (Šmarda and Bureš 2006), and can be distributed over nutrient availability and edaphic gradients in the field (Prentice et al. 2000; Fridley et al. 2011). Here, we used a long‐term climate manipulation study to examine whether 16 years of selection under summer drought treatment has selected for a shift in genome size in the grass *Festuca ovina* . In this long‐term experiment, drought has increased the abundance of species with traits associated with stress tolerance, relative to the control treatment (Fridley et al. 2016). In contrast, the control environment has higher species richness and higher levels of biomass productivity than the drought treatment (Grime et al. 2008), leading to a more competitive environment under conditions of nutrient limitation (Fridley et al. 2011). We predicted that if selection imposed by experimental drought operates in line with the genome streamlining hypothesis, then *F. ovina* individuals with large genome size should tend to occur in the drought treatment, since it is in this environment that the requirement for rapid growth under conditions of low nutrient availability is relaxed. We also examined the association between genome size and fine‐scale (within‐treatment) spatial heterogeneity in soil depth and edaphic conditions, and between genome size and plant phenotypes (cell size, flowering time and biomass). Our results do not support a role for drought in driving intraspecific differentiation in genome size, but instead suggest that deeper soils impose selection for *F. ovina* individuals with smaller genome size, and indicate that genome size may be under either direct or indirect stabilising selection in the field.

## 2 Materials and Methods

### 2.1 Study Site and Study Species

Our study uses a long‐term climate manipulation study at the Buxton Climate Change Impacts Laboratory (BCCIL), in Derbyshire. Here, climate treatments have been applied annually to 3 m × 3 m plots of intact calcareous grassland since 1993 (Grime et al. 2000). Treatments are replicated five times in a randomised block design, including control plots that experience the ambient climate. Here, we utilise plant material collected from drought‐treated and control plots. The drought treatment is applied annually during July and August using automatic rain shelters, which cover plots when it is raining and return to an off‐plot position once the rain stops. This treatment drives a significant reduction in soil water potential (−1100 kPa in drought plots vs. −20 kPa in control plots, at 5 cm depth; see figure 4 in Fridley et al. 2011). Significant cm‐scale soil depth heterogeneity occurs within plots (ranging from 0 to > 30 cm), such that the range in soil depth typifying the site is replicated within each plot (Fridley et al. 2011).

The genus *Festuca* is an established model system for studying intraspecific variation in genome size, which has been documented in *F. polesica*, *F. rupicola* , *F. vaginata,* and *F. pallens* (Šmarda 2006). *Festuca ovina* L. is a perennial grass with wind‐pollinated, self‐incompatible, hermaphroditic flowers typical of grasslands with low fertility (Ghatnekar 1999; Hill et al. 2004; Stace 2010; Weilbull et al. 1991). It is the most abundant and the most frequent plant species at BCCIL and has increased in cover in the drought plots relative to the control plots (Fridley et al. 2011). Population genetic data suggest that clonal growth in *F. ovina* at BCCIL is of limited demographic importance relative to sexual recruitment and supports the existence of genetic differentiation between drought and control treatments at the site (Ravenscroft et al. 2015; Trinder et al. 2020). DNA content data suggest that a tetraploid lineage of *F. ovina* is present on limestone grassland in the Derbyshire and Peak District Dales (Grime and Mowforth 1982).

### 2.2 Collection and Propagation of *F. ovina*

In July 2010, small bunches of 4–8 connected tillers of *F. ovina* were sampled from the drought and control plots at BCCIL following 16 complete years of drought treatments. Thirty individuals were collected from both the drought and control treatments (six individuals per plot, per treatment), using a spatially stratified sampling design, and soil depth was recorded at each sampling site (full details provided in Appendix A; Trinder et al. 2020). Plants were established in 3 L pots containing a 3:1 mix of John Innes No. 1 potting compost and medium‐grade Perlite (LBS Horticulture). The clonal lines were housed in purpose‐built raised ‘bays’ at Ness Botanic Gardens, University of Liverpool, UK. They were maintained by seed head removal during the summer, biomass clipping in September and supplementary watering as required. One individual failed to establish, leaving a total of 59 clonal lines. Hereafter, these field‐collected clonal lines are referred to as the parental or P1 plants. We established an F1 progeny array from the P1 plants during the flowering season of 2012 (May–June), via natural wind pollination. In total, 472 F1 plants were established, and these were maintained at Ness Gardens as clonal lines using the methods applied to the P1 clonal lines.

### 2.3 Flow Cytometry

In April 2014 and May 2015, we carried out two flow‐cytometry experiments at the Jodrell Laboratory, Royal Botanic Gardens, Kew. In the first, we screened all P1 and F1 individuals, with the aim of determining the ploidy of the experimental plants. The first flow‐cytometry experiment revealed significant variation in genome size among *F. ovina* individuals. Therefore, in the second experiment, we focused only on the P1 individuals and selected F1 individuals that possessed extreme values of genome size. In this latter experiment, we aimed (i) to validate the existence of variation in genome size within our study population, in both P1 and F1 plants and (ii) to make replicated, robust estimates of genome size for the P1 individuals that could subsequently be used to assess the potential adaptive value of genome‐size variation.

To support the first flow‐cytometry experiment, leaf samples from two living tillers of each of the P1 and F1 plants were collected in sealed Ziploc plastic bags containing wet tissue paper and stored at 4°C. A basal section of young leaf of each *F. ovina* sample was combined with *Petunia hybridum* ‘PxPc6’ leaf tissue as an internal standard (2C = 2.85 pg; Marie and Brown 1993), and chopped using a razor blade in a petri dish containing 1000 μL ‘General Purpose Buffer’ (GPB; Loureiro et al. 2007) supplemented with 3% PVP‐40. The mixture was filtered through a 30 μm nylon mesh (Partec), and then stained with 50 μL propidium iodide (Sigma, 1 mg mL^−1^) and left on ice for 15 min. Three thousand cells were analysed in each measurement on a PAII flow cytometer (Partec, Germany) equipped with a 100 W high‐pressure mercury lamp. DNA content was then calculated for each sample compared to the internal standard as follows: DNA content = (Mean peak of the sample/Mean peak of the standard) × 2C DNA content of the standard.

In the second flow‐cytometry experiment, we estimated the DNA content of the P1 lines and three selected F1 individuals. Methods followed those above, with the following amendments. For each sample, a basal section of young *F. ovina* leaf was chopped along with a *Petroselinum crispum* ‘Champion Moss Curled’ internal standard (2C = 4.5 pg; Obermayer et al. 2002). Nuclear DNA content was estimated by recording at least 1000 cells for both sample and internal control peaks on a Partec CyFlow Space flow cytometer (Sysmex‐Partec, UK) fitted with a 100 mW green solid‐state laser (Cobolt Samba, Solna, Sweden). Measurements took place over the course of 3 weeks, and samples were measured in a randomised order. Three replicate leaves were measured per P1 line, and a mean DNA content was calculated as the average of these three values. The existence of genome‐size variation among F1 plants was validated by co‐chopping tissues from different F1 individuals in a single measurement. We also estimated the repeatability of the replicate P1 genome‐size measurements arising from the second flow‐cytometry experiment (Appendix A.1). The repeatability of our genome‐size measurements, following Nakagawa and Schielzeth (2010), was 0.963 (this decreased to 0.702 when excluding one individual (‘2936’), the genome size of which was an outlier). Finally, we assessed the precision of individual genome‐size estimates by calculating the coefficient of variation (CV) in genome size among the assayed cells within samples. The average CV for the internal standard ( *Petroselinum crispum* ) was 2.52%, while that for the sample ( *Festuca ovina* ) was 2.18%. Genome‐size measurements were obtained for 57 of the 59 P1 plants. One plant, ‘2936’, had a much larger genome size than the other plants; therefore, this individual was excluded from the statistical analyses.

### 2.4 Chromosome Counts

The P1 and F1 plants with the largest and smallest genome sizes in the main range of variation (as measured using flow cytometry), along with any P1 or F1 plants with outlier genome‐size measurements, were selected for chromosome counting, to evaluate the contribution of variation in ploidy to differences in genome size. Chromosome counts were carried out on fresh, rapidly growing root tips using phase‐contrast microscopy (the full method, modified from Dyer 1963, is provided in Appendix A.2).

### 2.5 Phenotypic Measurements

Genome size has been shown to be associated with cell size, and this could be a mechanism underpinning the relationship between genome size and other plant phenotypes (Leitch and Leitch 2013). To study this, we measured cell size and two plant phenotype traits: flowering time and plant biomass. Guard cell length was selected as a measure of cell size, as this trait has only limited sensitivity to plant osmotic balance and cell turgor (Willmer and Fricker 1996). It is also the cell type in which genome size and cell size have previously been shown to correlate at the species level (Beaulieu et al. 2008). Guard cell length was measured using stomatal peels (methods in Appendix A.3). On average, 15 guard cells were measured for each leaf, and the mean guard cell length was calculated for each P1 line. No guard cell measurements could be obtained for one individual, so for this trait, the dataset was reduced to *N* = 55.

Flowering time and biomass data were taken from a common garden experiment in which clonal replicates of the P1 lines had been grown in a common environment at Ness Botanic Gardens (full methods in Trinder et al. 2020; Section 2.6). Flowering time data were collected during the summer of 2013. Flowering time was measured as the date of first anthesis (at which the first anther is visible in any spikelet). Biomass was measured by cutting all vegetative biomass 25 mm above the soil surface in September 2013; it was then dried for 1 week at 55°C and weighed. Dry biomass was the mean of four or five replicates for each clonal line.

### 2.6 Statistical Analysis

We used linear mixed models (LMMs, with Gaussian error distributions) to examine the relationship between intraspecific variation in genome size (using the mean value for each individual), and environmental and phenotypic variables. Models were fitted in R (R Development Core Team 2025) using the package mcmcglmm (Hadfield 2010). To test for relationships between genome size and climate treatment and mean soil depth at BCCIL, we used genome size as a response variable, and climate treatment and soil depth as fixed effects. To assess associations between guard cell size and climate treatment and genome size, we modelled guard cell size as a response variable with climate treatment and genome size as fixed effects predictors. Finally, we examined whether either plant dry biomass or flowering time, as response variables, were associated with genome size, as a fixed effect predictor. Biomass data were square root transformed prior to analysis. We fitted an experimental block at BCCIL in all of the models above, as an additional fixed effect (see Appendix A.4 for model specifications). Finally, to examine whether variation in genome size differed between deep and shallow soils, we performed a Levene's test for equality of variances. Shallow and deep soils were identified with reference to the median soil depth for the dataset.

## 3 Results

### 3.1 Intraspecific Variation in Genome Size

**FIGURE 1:**
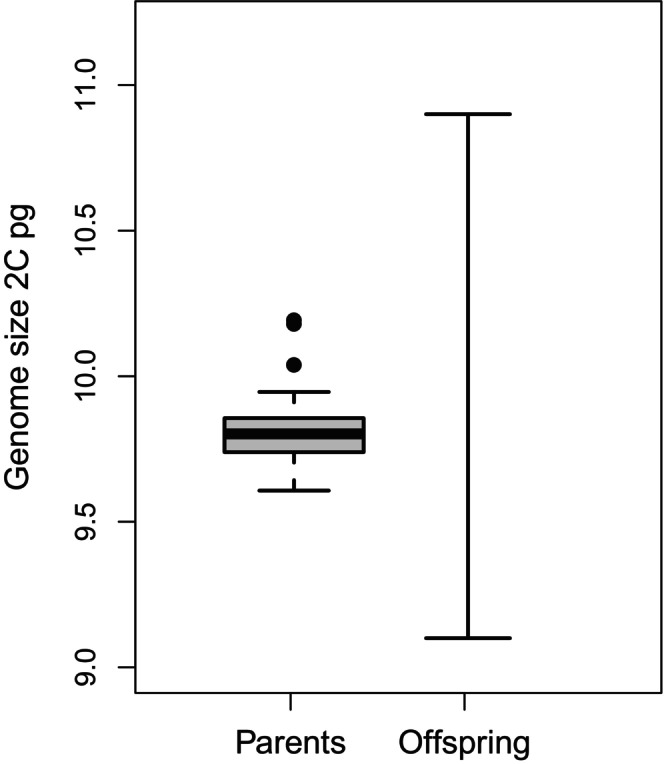
Genome‐size variation in *Festuca ovina* parent plants (P1) and their offspring (F1). The plot excludes data for an outlier P1 plant (‘2936’) and a hexaploid offspring individual (‘2268‐B3’). The boxplot for the offspring plants comprises data from only two individuals, hence no box is shown. The box shows the interquartile range, the bar shows the median value and the whiskers extend out to the most extreme data point that is no greater than 1.5× the interquartile range from the edge of the box.

**FIGURE 2:**
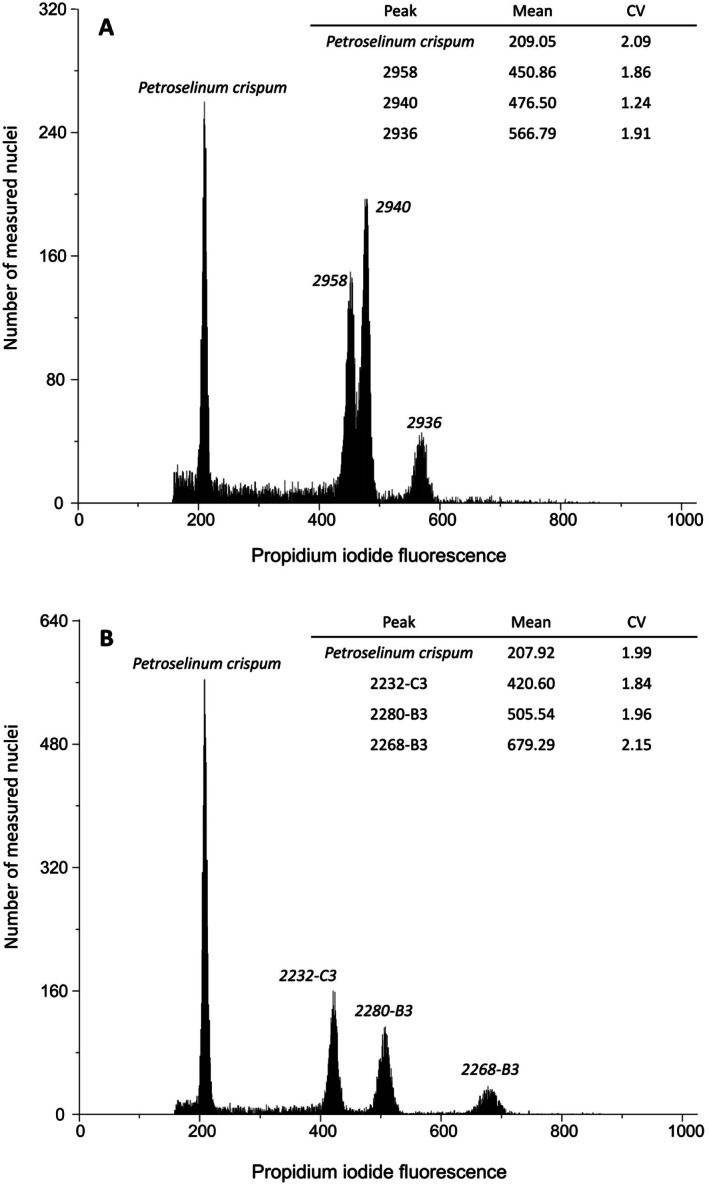
Differences in DNA content in simultaneously measured samples of *F. ovina* and a standard of *P. crispum* . Inset tables give mean location and coefficient of variation (CV) of DNA content for each individual included in the plot (individual plants are identified in the ‘Peak’ column). (A) Maximal differences among P1 (parental) *F. ovina* plants (three individuals are shown along with a *P. crispum* standard). (B) Maximal differences among F1 (offspring) *F. ovina* plants (three individuals are shown along with a *P. crispum* standard).

In total, ploidy was determined for 450 F1 offspring and 57 P1 parent plants in *F. ovina* , of which 449 F1 offspring and 57 P1 plants were identified as tetraploids (4*x*). The average 2C genome size of the tetraploid P1 plants was 9.85 pg (range 9.61–12.28 pg), representing a 1.278‐fold maximum difference in genome size among individuals. However, one P1 individual, ‘2936’, also had a considerably larger 2C genome size than the rest of the P1 plants (12.28 pg). The mean genome size of P1 plants excluding the outlier was 9.81 pg (range 9.61–10.19), a maximum 1.061‐fold among‐individual difference in genome size (Figures 1 and 2A). Chromosome counts supported the P1 outlier as being a tetraploid and did not identify any extra chromosomes. The parent plant with the largest genome size, excluding the outlier, was tentatively identified as aneuploid (2*n* = 26) by chromosome counting (Table A1). One offspring individual, ‘2268‐B3’, was identified as having a very large 2C genome size, 14.70 pg, consistent with it being a hexaploid (6*x*). This was supported by its chromosome count 2*n* = 42 (chromosome counts are given in Table A1). Excluding the hexaploid individual, a maximum 1.202‐fold difference in genome size was observed between the F1 individuals with the highest and lowest genome‐size estimates (Figures 1 and 2B). The F1 plants had a wider range of intraspecific variation in genome size than the P1 plants, with the hexaploid and outlier parent individual excluded (see Figure 1). Neither of the individuals with larger genome sizes differed morphologically from the remaining tetraploid plants.

### 3.2 Variation in Genome Size With Abiotic Environment

**FIGURE 3:**
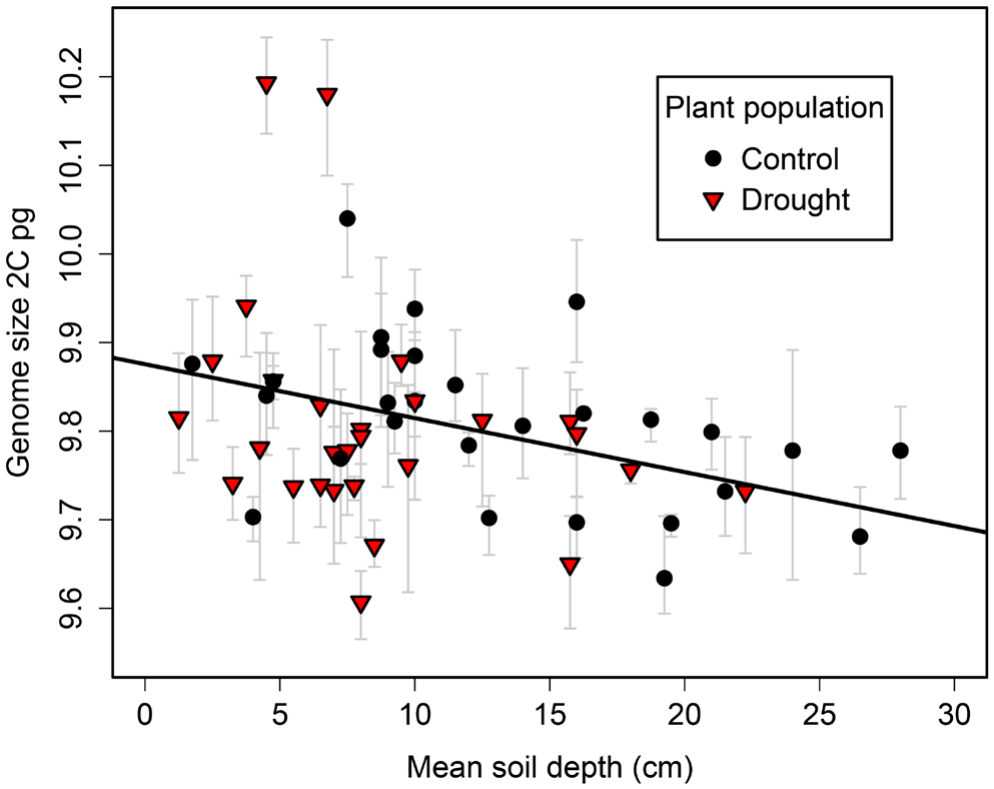
Genome size of *F. ovina* individuals collected from BCCIL is predicted by the soil depth of their growing locations in the field. ‘Plant population’ indicates the climate treatment from which the plant originated. Plot shows data for P1 (parental) *F. ovina* plants. Error bars give minimum and maximum estimates of genome size for each plant.

There was no significant difference in the genome size between P1 plants from the drought and control treatments at BCCIL (mixed effects model; pMCMC = 0.956, *n* = 56). However, there was a significant negative association between genome size and soil depth (mixed effects model; pMCMC = 0.022, *n* = 56; Figure 3), implying that individuals with large genomes were more likely to be found in shallow soils than individuals with small genomes. Two individuals with large genomes appeared to be driving this pattern. However, genome size was still negatively correlated with soil depth when these two individuals were excluded from the analysis (mixed effects model; pMCMC = 0.066, *n* = 54). There was no significant difference in the among‐individual variance in genome size between P1 plant sub‐populations from shallow and deep soil (Levene's test; *F* = 2.441, *p* = 0.124, *n* = 56).

### 3.3 Genome‐Size Phenotype Associations

**FIGURE 4:**
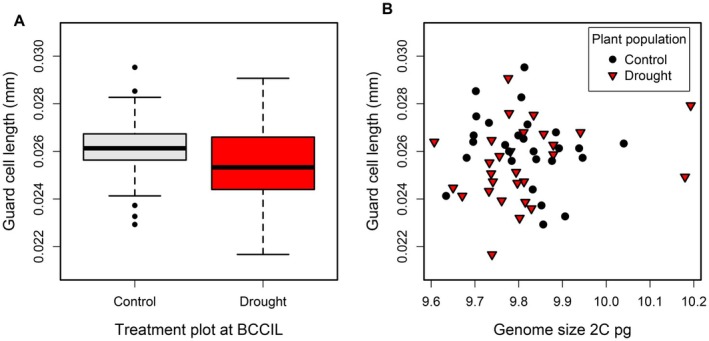
*F. ovina* guard cell length is associated with neither climate treatment (plant population) nor genome size. (A) Boxplot summarising the distribution of population guard cell lengths in drought and control climate treatments at BCCIL. The box represents the first and third quartiles, whiskers represent ±1.5× interquartile range, with points lying outside the range of the whiskers being outliers. (B) Relationship between plant genome size and mean guard cell length. Both plots show data for P1 (parental) *F. ovina* plants.

The average guard cell length was 25.8 μm. There was no significant difference in average guard cell length between drought and control treatments at BCCIL (mixed effects model; pMCMC = 0.146, *n* = 55; Figure 4A). There was no significant association between genome size and any of the plant traits that we measured (guard cell size, flowering time and dry biomass; mixed effects models pMCMC > 0.05; Figure 4B).

## 4 Discussion

In this study we used a chronic climate manipulation experiment to test whether 16 years of selection under drought had altered genome size at the population level in *Festuca ovina* . We have also assessed the relationships between genome size and soil depth, and genome size and phenotype to determine whether genome‐size variation has adaptive value in a changing climate. Our results revealed the existence of substantial intraspecific variation in genome size within our *F. ovina* study population at BCCIL (1.278‐fold). We have found no evidence that chronic drought treatment had selected for differences in genome size between the drought and control treatments. However, our results did provide evidence that plant genome size is associated with fine‐scale differences in soil depth that occur within our study site. Finally, our results show that (with the exception of an outlier) the range of genome size in F1 offspring plants is larger than in the field population (P1 parent plants). Together, our results suggest that either genome size itself or a correlated trait is under selection. Plants growing on shallower soils at BCCIL had larger genome sizes. However, we have not, as yet, identified any association between genome size and plant phenotypes, and therefore, the trait or mechanism that might mediate this relationship is unknown.

### 4.1 Environmental Variation and Genome Size

We have found no evidence that long‐term drought treatment at BCCIL has selected for altered genome size in *F. ovina* . In a comparable study, Pellicer et al. (2010) investigated differences in genome size in six plant species at a long‐term climate change experiment in the Garraf Massif, Catalonia. They found no significant differences in genome size between warming, drought or control treatments, following 7 years of treatment. Our study more than doubles the length of time under climatic selection and found no differences in genome size between drought and control treatments. Given the range of variation we observe in the offspring data, this is not due to lack of variation in genome size. We note, though, that the persistence of long‐lived individuals that predate the start of treatments at BCCIL could complicate the interpretation of this result. If climatic selection acting on plant genome size occurs at the plant establishment phase (during recruitment), and selection acting on already‐established individuals is weak, then the persistence of individuals that predate the experiment will weaken the power to detect an effect of the drought treatment on genome size. However, our past genetic and phenotypic data from BCCIL suggest that clonal growth makes only a limited contribution to population structure, relative to sexual recruitment, implying that the frequency of individuals predating the onset of the climate treatments is likely to be low (Ravenscroft et al. 2015; Trinder et al. 2020). Our results, therefore, add to the evidence that drought exerts little or no selection pressure on plant genome size, either directly or indirectly via genetically correlated phenotypes.

We have found that genome size is negatively correlated with soil depth, which varies over a fine spatial scale (tens of centimetres) in our study system. This result suggests that the edaphic environment can shape variation in plant genome size, either directly or through genetically correlated traits. At BCCIL, soil depth covaries with many biotic and abiotic variables. These include pH, moisture availability (in the drought treatment), plant N supply, above‐ground plant biomass production, the abundance of different coexisting plant species and the competitive environment (Fridley et al. 2011, 2016). We suggest that variation in soil moisture associated with soil depth is unlikely to explain the association between genome size and soil depth, since we did not observe any significant difference in genome size between the drought and control treatments.

At BCCIL, soil nitrogen availability is not significantly associated with soil depth, but the highest nitrogen availability is found on the shallowest soils (Fridley et al. 2011). Larger genomes require greater quantities of nitrogen and phosphorus, because nucleic acids are highly demanding of these two nutrients (Hessen et al. 2010; Guignard et al. 2016). Long‐term nutrient addition experiments have shown that nutrient addition treatments tend to favour plant species with larger genome sizes (Guignard et al. 2016; Šmarda et al. 2013). Soil nutrient availability could, therefore, be an important factor through which genome size can constrain the distribution of different plant species. We suggest that localised high nitrogen availability in shallow soils at BCCIL may weaken selection against individuals with large genomes and permit these individuals to persist.

Differences in competitive intensity across the soil depth gradient at BCCIL could also be responsible for the relationship between genome size and soil depth that we observed. Above‐ground biomass production at BCCIL increases with soil depth up to 18 cm (Fridley et al. 2011). The greater biomass supported by deeper soils at BCCIL may lead to a more intense competitive environment in these edaphic microhabitats. Šmarda et al. (2010) demonstrated that competition can be an important selective force on intraspecific variation in genome size. In populations of *F. pallens* raised under high levels of competition, individuals with the largest and smallest genomes were selected against, resulting in lower genome‐size variation compared to populations raised without competition. A relaxation in the intensity of competition on shallow soils at BCCIL may allow plants with larger genomes to establish without being selected against and removed. The effects of nutrient availability and competition could act together to exert selection on plant genome size. On the shallowest soils, the higher localised nutrient availability and reduced competition may permit the successful establishment of *F. ovina* plants with large genomes, whereas on deeper soils, there is selection for smaller genome size. Further work on this population will be required to (i) elucidate the ecological factors imposing selection on genome size in *F. ovina* at BCCIL and (ii) identify the phenotypic and molecular mechanisms through which genome size is associated with fitness. We suggest that analyses of below‐ground traits (including clonal and root architecture and root traits), which we did not investigate in this study, might be particularly valuable in understanding how plant phenotypes mediate the relationship between genome size and adaptation to edaphic conditions.

Beyond BCCIL, associations between intraspecific genome size and latitude, longitude, altitude and microhabitat have been found in a number of studies (Bureš et al. 2004; Duchoslav et al. 2013; Kalendar et al. 2000; Šmarda and Bureš 2006), suggesting that abiotic and biotic environmental conditions may impose selection on genome size (directly or indirectly). However, the observational nature of these studies precludes the identification of specific environmental variables that are causally linked with genome size. Our study, and that of Pellicer et al. (2010), used replicated experimental treatments with respect to drought. Both studies have failed to find a significant difference in genome size between drought and control plots across a total of seven species. This suggests that drought is unlikely to be the environmental variable driving the observed associations between genome size and habitat seen in other studies.

At the species level, plant genome size is weakly associated with responses of species to drought treatment at BCCIL (Fridley et al. 2016). Responses of species to drought were positively (but non‐significantly) associated with plant genome size on shallow soils (*r* = 0.305) but not on deep soils (*r* = 0.082). Fridley et al. (2016) also used co‐inertia RLQ analysis to show that drought and genome size were positively correlated at the community level. However, DNA content had a low importance in comparison with the other traits studied (9th out of 18 traits included in the analysis). These results reinforce the conclusion that drought has only a weak relationship with genome size in plants, at both the intraspecific and interspecific levels. That the weak association between genome size and plant species responses to drought was restricted to shallow soils suggests, in addition, that such a relationship is most likely to occur in conditions where selection is relaxed, either through lower levels of competition or increased resource availability (Fridley et al. 2011, 2016).

### 4.2 Phenotypic Target of Selection

Our results are consistent with those of other studies in suggesting that aspects of the abiotic or biotic environment can exert selection on intraspecific variation in genome size. However, it is unclear whether genome size itself is the target of selection or whether other correlated plant phenotypes mediate the effects of selection on genome size. Correlations between phenotypes and genome size tend to be weaker within plant species than between plant species (Greilhuber and Leitch 2013). Intraspecific relationships between genome size and plant traits have been found for the rate of seedling growth (Šmarda et al. 2008) and plant flowering date (Benor et al. 2011). Other studies have failed to find a relationship between plant traits and genome size within plant species (Pavlíček et al. 2008; Šmarda and Bureš 2010). In the present study, we found no evidence that genome size was correlated with any of the phenotypic traits measured: guard cell size, flowering time, or vegetative biomass. Thus, we are unable to comment on whether soil depth variation exerts either direct or indirect selective pressure on genome size.

### 4.3 Mechanisms of Change in Genome Size

Genome size increase is typically the result of polyploidisation and the accumulation of transposable elements, whereas genome‐size reduction is the result of illegitimate recombination and intra‐strand homologous recombination (Hawkins et al. 2008; Hu et al. 2011). Transposable elements are known to form a large fraction of grass genomes (Devos 2010; Li et al. 2004; Messing et al. 2004) and can also be ecologically adaptive through the creation of insertional polymorphisms (Wendel and Wessler 2000). For example, in a study on *Hordeum spontaneum* , Kalendar et al. (2000) showed that the copy number of the retrotransposon *BARE*‐1 is positively correlated with plant genome size, and is also associated with the most arid sites. These findings suggest that specific transposable elements are likely to be candidates in driving variation in genome size in *F. ovina* , given their prevalence and activity in grass genomes (Devos 2010). We have also identified a single offspring individual ‘2268‐B3’ as a hexaploid (6*x*). This individual appears to illustrate the spontaneous generation of a hexaploid, as all of the parent plants were identified as tetraploids (Hornsey 1973; Ramsey 2007). We suggest that this is the result of irregular gametogenesis, resulting from the combination of a reduced 2*x* gamete with an unreduced 4*x* gamete (Ramsey 2007; Ramsey and Schemske 1998). Furthermore, one of the parent individuals was identified as a putative aneuploid (2*n* = 26; Table A1). The existence of variation in ploidy and, possibly, also chromosome number could provide an alternative or additional explanation for the intraspecific variation in genome size that we observed. However, given that our genome‐size data were derived from leaves, and our chromosome counts from roots, a comparison of genome size in roots and leaves should be undertaken for the individuals with extreme genome sizes, along with additional chromosome counts, to validate the contribution of variation in chromosome number to intraspecific genome‐size variation.

### 4.4 Variation in Genome Size Between Generations

We identified a greater range of genome size in F1 offspring than in parent plants (parents = 1.061‐fold; offspring = 1.202‐fold). This result corresponds closely with those of Šmarda et al. (2008) who also found that *F. pallens* offspring plants have a greater range in genome size than their field‐collected parents. Later, Šmarda et al. (2010) demonstrated that this pattern was the result of stabilising selection removing the smallest and largest genomes. Both our study and that of Šmarda et al. (2008) demonstrate that considerable variation in genome size can be generated in a single sexual generation. We propose that in the field, excess variation in genome size is generated in each new sexual generation of *F. ovina* , which Šmarda et al. (2008) suggest could be the result of segregation acting on standing variation in chromosome size. We suggest that stabilising selection then acts during establishment, to remove the larger and smaller genomes, reducing variation in the established phase of the plant life cycle. The presence of the outlier individual ‘2936’ in the natural population suggests that this force is weak in our study system, and that individuals with extreme genome sizes may occasionally establish in the wild (Beaulieu 2010).

## 5 Conclusions

We have documented a negative relationship between genome size and soil depth, but we found no evidence that genome size has responded to selection imposed by long‐term drought treatment at BCCIL. Genome size was not associated with plant phenotypes measured in a common garden experiment (cell size, flowering time and biomass), and the target of selection remains unknown. We propose that locally relaxed selection pressures in shallow soils, combining both higher nutrient availability and reduced competition, may allow individuals with larger genome sizes to persist. We have also shown that the potential range of genome size in offspring is larger than that in the established population in the field, which suggests that stabilising selection is acting to remove larger and smaller genomes during plant establishment. Together, our results suggest that genome size is both variable and under natural selection within *F. ovina* populations, but we do not yet know whether selection is either direct or indirect, or which edaphic conditions are shaping genome‐size variation.

## Author Contributions

**Sarah Trinder:** conceptualization (equal), data curation (equal), formal analysis (lead), investigation (lead), methodology (equal), project administration (equal), validation (equal), visualization (lead), writing – original draft (lead), writing – review and editing (lead). **Oriane Hidalgo:** investigation (equal), methodology (equal), supervision (supporting), validation (equal), writing – review and editing (equal). **Michael F. Fay:** conceptualization (equal), funding acquisition (equal), supervision (equal), writing – review and editing (equal). **Andrew P. Askew:** investigation (supporting), methodology (supporting), resources (equal), writing – review and editing (equal). **Hugh McAllister:** investigation (equal), writing – review and editing (equal). **Raj Whitlock:** conceptualization (equal), data curation (equal), funding acquisition (lead), investigation (supporting), methodology (equal), project administration (equal), resources (equal), supervision (equal), writing – review and editing (equal).

## Conflicts of Interest

The authors declare no conflicts of interest.

## Data Availability

The data archive and code needed to repeat the analyses described in this paper are available on Dryad (https://doi.org/10.5061/dryad.2rbnzs7tt).

## Appendix A

### Genome‐Size Measurement Repeatability

Each P1 genome‐size measurement is an average of three measurements of genome size for each P1 plant. The repeatability of the genome‐size measurements was calculated using a linear mixed effects model, in the package LMER (Bates et al. 2015). The dataset consisted of each of the three genome‐size measurements for each clonal line. Clone was fitted as a random effect. This apportioned the variance in genome size into that which was within P1 clonal lines and that which was among P1 clonal lines. The repeatability was then calculated as:

R=Between clone variance/Total variance

following (Nakagawa and Schielzeth 2010). The repeatability of genome‐size measurement was *R* = 0.963, which decreased to *R* = 0.702 when measurements for the outlier individual 2936 were removed from the dataset.

### Chromosome Counting Methods

Chromosome counts were carried out by Dr Hugh McAllister. The *Festuca ovina* individual with the largest and smallest genome sizes in the main range of variation (as measured using flow cytometry), along with individuals with outlier genome‐size measurements, was selected for chromosome counting, for P1 and F1 plants. Chromosome counts were conducted using a method modified from Dyer (1963). Chromosome counts were conducted on rapidly growing root tips. Root tips of ~1 cm length are removed with forceps and placed in vials of 1‐bromo‐naphthalein solution at room temperature for 4 h. Vials were placed in a fridge overnight at ~1°C. Root tips were fixed in a solution of 3:1 ethanol (95%):glacial acetic acid for 12 h at −18°C. Root tips were removed from the vials and transferred into preheated vials of 1 M HCl at 60°C for 5 min. After hydrolysis, root tips were placed in 70% ethanol and stored for at least 12 h. The root tip was then placed in a drop of 70% ethanol on the edge of a slide and examined under a dissecting microscope. The root cap was removed using fine needles, and the densely cytoplasmic region was removed. This was placed in a drop of mounting solution of 2:1 lactic acid:propionic acid, in the centre of the slide and a coverslip was added. The slide was tapped with a needle to produce a monolayer of cells. The slide was then blotted to remove excess mounting solution and squashed between absorbent paper. The preparation was examined using phase‐contrast microscopy.

### Guard Cell Length Phenotypic Measurements

The longest leaf on each plant was collected, placed in a plastic bag with wet tissue paper to maintain leaf moisture content, and stored in a fridge prior to processing. Each leaf was cut in half along its length under a dissection microscope, allowing the inside edge of the leaf, where the stomata are located, to be visible. The inside edge of the leaf was painted with clear nail varnish and left to dry for 5 min. A piece of Sellotape was placed on top of the nail‐varnished area and quickly peeled off, transferring the nail varnish imprint onto the Sellotape. This was then stuck to a microscope slide and viewed at 40× magnification on a UNILUX‐12 light microscope (Kyowa Optical Co. Ltd). A picture was taken using a Canon ESO 1000D digital SLR camera mounted to the microscope. Images of the guard cells were analysed using ImageJ (Abramoff et al. 2004). Measurements were calibrated against an image of a stage micrometre. The lengths of, on average, 15 guard cells were measured for each leaf, and an average guard cell length was calculated for each plant.

### LMM Model Specifications

We used linear mixed models (LMMs, with Gaussian error distributions) to examine the relationship between intraspecific variation in genome size and environmental and phenotypic variables. Models were fitted in R (R Development Core Team 2025) using the package mcmcglmm (Hadfield 2010). mcmcglmm uses a Bayesian approach to implement models. Unless specified otherwise, LMMs had the following specifications. Models were run for 1,300,000 iterations, with a burn‐in of 300,000 and a thinning interval of 1000, resulting in a sample size of 1000. The prior for variance components was a non‐informative uniform improper prior distribution on the standard deviation of the random effects (specified as *V* = 1.0 × 10^−16^, nu = −1 in mcmcglmm), as recommended by Gelman (2006). For each model, the sensitivity to starting parameters was tested by running the model three times with over‐dispersed chain starting values using the parameter *start=list(QUASI=FALSE)*, and using the Gelman‐Rubin diagnostic to assess convergence (Gelman and Rubin 1992). The blocking factor for plots at BCCIL was fitted as a fixed effect (five levels) in models, and contrasts for the plot block variable were centred to allow estimation of the remaining parameters to a notional ‘average’ block. We used the *autocorr* function to check the autocorrelation, which is the level of non‐independence between successive samples of the chain. The autocorrelation between the first and second successive stored samples was checked, and values below 0.15 were required for the model to be accepted, following the recommendation of Hadfield (2012). Post hoc comparisons for particular parameter contrasts were made by re‐levelling variable levels as needed. We report ‘95% credible intervals’ which is the range within which we expect, with a probability of 0.95, the true parameter value to be located. Credible intervals were calculated using the *HPDinterval* function in mcmcglmm.

**TABLE A1:** Chromosome counts. Individuals of *F. ovina* on which chromosome counts were carried out, the resulting count, and the reason that individual was selected. All selections were based on the first flow‐cytometry screening experiment.

Individual	Count	Description, reason for selection
2958	28 (26)	Parent plant identified with the smallest genome size
2940	26	Parent plant identified with the largest genome size, except for the outlier individual
2936	28	Parent plant identified with the outlier genome size
2232‐C3	28	Offspring plant identified with the smallest genome size
2280‐B3	28	Offspring plant identified with the largest genome size, except for the putative hexaploid.
2268‐B3	42	Offspring plant identified with the largest genome size, the putative hexaploid
